# Broad lytic spectrum of novel *Salmonella* phages on ciprofloxacin-resistant *Salmonella* contaminated in the broiler production chain

**DOI:** 10.14202/vetworld.2022.2039-2045

**Published:** 2022-08-23

**Authors:** Wattana Pelyuntha, Arsooth Sanguankiat, Attawit Kovitvadhi, Kitiya Vongkamjan

**Affiliations:** 1 Department of Biotechnology, Faculty of Agro-Industry, Kasetsart University, Bangkok, 10900, Thailand; 2 Department of Veterinary Public Health, Faculty of Veterinary Medicine, Kasetsart University, Kamphaeng Saen, Nakhon Pathom, 73140, Thailand; 3 Department of Physiology, Faculty of Veterinary Medicine, Kasetsart University, Bangkok, 10900, Thailand

**Keywords:** antibiotics, bacteriophage, fluoroquinolone, phage lysis, poultry

## Abstract

**Background and Aim:**

Ciprofloxacin (CIP) is recommended for salmonellosis treatment as the drug of choice; however, overuse of this drug can cause drug resistance issues and failure to treat diseases. Phage therapy is an alternative approach for combatting CIP-resistant infection. This study aimed to estimate the prevalence of CIP-resistant *Salmonella* isolated from the broiler production chain and evaluated the lytic ability of novel *Salmonella* phages isolated from water samples.

**Materials and Methods:**

Samples were obtained from the broiler production chain and used for *Salmonella* isolation. serovar and CIP resistance of each isolate were characterized through latex agglutination and agar disk diffusion test, respectively. Water samples from different sources were acquired for phage isolation. The lytic activity of novel-isolated phages was also examined.

**Results:**

In this study, 51 *Salmonella* isolates were recovered from the broiler production chain (two commercial farms, one free-range farm, two slaughterhouses, and three stalls from the wet market). Kentucky was the major serovar characterized (16), followed by Typhimurium (9), Agona (5), Corvalis (5), Schwarzengrund (5), Singapore (3), Weltevreden (3), Mbandaka (2), Give (2), and Albany (1). The serovars that exhibited CIP resistance were 14/16 isolates of serovar Kentucky (87.5%) and one isolate of serovar Give (50%), whereas eight other serovars were susceptible to this drug. Overall, the prevalence of CIP-resistant *Salmonella* recovered from the sources included in this study was 29.4%. This study identified 11 *Salmonella* phages isolated from wastewater samples derived from broiler farms, wastewater treatment stations, and natural reservoirs. Our phages showed the total percentage of lysis ability ranging from 33.3% to 93.3% against CIP-resistant isolates. However, only one bacterial isolate, namely 210SL, recovered from the food contact surface of a wet market stall and was resistant to all phages.

**Conclusion:**

Diverse serovars of *Salmonella* were recovered in the broiler production chain in this study, while the isolates presenting CIP-resistant *Salmonella* were as high as 29.4%. Overall, *Salmonella* phages showed high lysis ability against these CIP-resistant *Salmonella* isolates, suggesting the potential application of phage-based treatments or biocontrol in the broiler production chain.

## Introduction

Antibiotic resistance in food-borne bacterial pathogens is a significant concern for public health safety worldwide through the misuse of antibiotics in food and animal production systems. Antibiotic-resistant bacteria can be spread to humans through the food supply chain [1, 2]. The infection with antibiotic-resistant bacteria causes a serious illness and failure to treat diseases with regular treatment of the usual antibiotics. In the United States, food-borne illnesses caused by antibiotic-resistant bacteria affect 2.8 million cases and 35,000 deaths annually [3].

*Salmonella* is one of the most reported pathogenic bacteria associated with human illness. The infection can be linked to consuming contaminated food and water. Animals, particularly poultry, are the primary carriers of this bacterium [4], and their products are associated with massive outbreaks [5]. Therefore, antibiotic resistance in *Salmonella* is a considerable threat to public health and food safety. At present, *Salmonella* has been reported to be resistant to many antibiotics [1, 6]. Some *Salmonella* strains showed resistance to at least one drug in three or more antimicrobial categories, showing multidrug-resistant (MDR) strains. The incidence of those is continually increasing worldwide [7]. Ciprofloxacin (CIP) is a member of the fluoroquinolone drug class used to treat numerous bacterial infections, especially *Salmonella* infection [8]. It inhibits bacterial DNA topoisomerase and DNA-gyrase activities during DNA replication [9]. Ciprofloxacin is classified as critically vital for human medicine by the World Health Organization [10]. This drug exhibits a broad spectrum of activity against Gram-negative bacteria such as *Escherichia coli*, *Klebsiella pneumoniae*, *Proteus mirabilis*, and *Pseudomonas aeruginosa* [11]. Still, it is less effective against Gram-positive bacteria such as *Staphylococcus aureus* and *Streptococcus pneumoniae* [9, 12]. Ciprofloxacin is recommended for salmonellosis treatment as a drug of choice; however, overuse of this drug can cause drug resistance issues, failure to treat diseases, and subsequent severe clinical outcomes [13]. Ciprofloxacin resistance in *Salmonella* has been increasingly reported worldwide because of plasmid-mediated-quinolone resistance in bacterial plasmids or chromosomes [14].

Bacteriophages or phages are viruses that can specifically kill bacteria and are not harmful to human and animal health. Phages are widely used to treat various pathogenic and MDR bacteria with strong bactericidal effects and high specificity [15]. In addition, phages can be used as an alternative approach for combatting CIP-resistant *Salmonella* [16].

Therefore, this study aimed to estimate the distribution of *Salmonell*a and CIP-resistant *Salmonella* in the broiler production chain and assess the lytic ability of novel *Salmonella* phages on CIP-resistant *Salmonella* recovered from this source. Data from this study will be helpful in designing phage-based therapies for controlling the widespread CIP-resistant Salmonella in the broiler production chain to resolve the harmful effects of antibiotic resistance threats on human and animal health.

## Materials and Methods

### Ethical approval

The study was not conducted on humans or animals and hence, it does not require ethical approval.

### Study period and location

The study was conducted from October 2021 to March 2022. Samples were collected from commercial broiler farms, free-range farms, slaughterhouses, and retail markets in Hat Yai city, Songkhla province, Thailand.

### Sampling location and collection of samples

This study included two commercial broiler farms, one free-range farm, two slaughterhouses, and three stalls from a wet market in southern Thailand. Two commercial farms are enclosed using an evaporative cooling system for animal cultivation (>24,000 broilers/cultivation cycle). Both farms are far from main roads, natural reservoirs, and human communities. These farms have adequate biosecurity measures for controlling straying insects and animals and highly hygienic conditions. These farms have their wastewater treatment ponds, and all dried waste was eliminated by incinerating or discarding it in a waste dump. Bedding material (rice husk) was packaged in the animal houses before being sold as manure. The free-range farm is not enclosed but far away from main roads and natural reservoirs. This farm contains about 500 birds/cultivation cycle. Two slaughterhouses are situated near main roads with high infrastructure and biosecurity measures to control straying insects and animals and are located far away from natural reservoirs. Waste was eliminated by incineration, and the wastewater was previously treated in its own small wastewater treatment ponds before being released into the environment. Three chicken meat stalls from a big wet market in Hat Yai city are not enclosed and are located near main roads and human communities. Wastewater and sewage were drained using the market drainpipe before integrating with other wastewater and sewage in the wastewater treatment canal of the city.

In commercial and free-range broiler farms, animal feed, boot cover swabs, cooling pad water, drinking water, soil, and bedding material (rice husk) were collected and acquired for *Salmonella* isolation. In slaughterhouses, food contact surfaces (working table, knife, cutting board, basket, and bucket), nonfood contact surfaces (wall and floor), and equipment (conveyor) surfaces were obtained through swabbing using a sterile cotton swab and kept in transported a liquid medium. For stalls at the wet market, chicken meat (about 200 g) was collected in sterile plastic bags. Food contact surfaces (stall surface, cutting board, knife, and ice bucket) and nonfood contact surfaces (wall and floor) were also obtained through swabbing, as previously described by Sripaurya *et al*. [17]. All samples were kept in an ice box (4°C) during delivery to the laboratory for analysis.

### Isolation and confirmation of *Salmonella* from samples

For *Salmonella* isolation, obtained samples were handled according to the protocol provided by the Biomérieux company (modified ISO 6579:2002). Approximately 25 g or 25 mL of animal feed, cooling pad water, drinking water, soil, bedding material, and chicken meat were mixed with 225 mL buffered peptone water (BPW, (Biomérieux, Marcy l’Étoile, France), and one tablet of *Salmonella* Supplement (Biomérieux) was added to inhibit undesired microorganisms. In addition, a 90 mL BPW and *Salmonella* Supplement Tablet was added to the boot cover and cotton swab samples. Then, all samples were incubated at 41.5°C for 18 h. *Salmonella* presented in the enriched samples was taken using streaking on a SALMA^®^ plate (Biomérieux) and incubated at 37°C for 24 h. A suspected colony (typical pink to purple) presented on the plate was further selected and restreaked on a tryptic soy agar plate to get a single colony. The stocks of each isolate were kept at −20°C in a 15% glycerol solution for further investigation. Then, single colonies were submitted for serotyping by a commercial service company (S&A Reagents Lab. Ltd., Part., Bangkok, Thailand) using a latex agglutination test.

### Characterization of CIP resistance profile in *Salmonella*

The CIP resistance test was examined with *Salmonella* isolated from positive samples (51 isolates). A standard agar diffusion test was conducted following the Clinical and Laboratory Standard Institute guidelines [18]. Isolates were propagated in tryptic soy broth (TSB) (HiMedia Laboratories, India) at 37°C overnight. The cell density of each culture was adjusted using TSB to attain an optical density (OD_625_) between 0.08 and 0.1. The diluted culture was swabbed onto Mueller-Hinton agar (MHA; Oxoid) using a sterile cotton swab. The CIP disk (5 mg, Oxoid Ltd., PW, UK) was placed on the center surface of the MHA plate. All plates were incubated at 37°C for 18 h to observe the inhibitory zone. Results were interpreted and recorded as sensitive, intermediate, and resistant according to the standard guideline. Two independent replicates were conducted [17, 18].

### Collection of water samples

Five wastewater samples from a broiler farm located in Bang Klam city, Songkhla Province, Thailand (W1), a wastewater treatment pond of the student dormitory of Prince of Songkla University (PSU) (W2 and W3), and a wastewater treatment pond at PSU hospital (W4 and W5) were obtained and used for phage isolation. One water sample from a natural reservoir (NR) in Hat Yai city, Songkhla, Thailand was also obtained. Approximately 500 mL of each sample was collected in a 1 L sterile white bottle and kept in an icebox (4°C) during delivery to the laboratory for further analysis.

### Isolation, purification, and preparation of *Salmonella* phages

The enrichment isolation method was used for *Salmonella* phage isolation utilizing the mixture of three *Salmonella* strains in our laboratory culture collection (*Salmonella* Agona H2-016, *Salmonella* Anatum A4-525, and *Salmonella* Kentucky S1H28). Enrichment and isolation steps were conducted following a principal protocol from our laboratory [15]. Plaques were evaluated on the bacterial host lawn, and the plaque morphotype was measured using a Vernier caliper. Every single plaque was selected for further purification thrice with the given host using a double-layer agar method. Purified plaque in salt magnesium (SM) buffer was ten-fold diluted to manufacture the semiconfluent lysis plates. A 5 mL SM buffer was added to each plate. All plates were orbitally shaken at 50 rpm at 25 °C for 2 h and then the buffer was obtained, and a top layer of agar was scrapped into 15 mL conical tubes. The supernatant was obtained by centrifugation at 6000 rpm for 15 min at 4°C, followed by filtration through 0.20 mm syringe filters before storing at 4°C. The titer of phages was counted using the double overlay technique [15].

### Determination of phage lytic ability

The lytic ability for each isolated phage was performed using a spot test on the given CIP-resistant *Salmonella* isolates. Briefly, 20 mL of each phage lysate was dropped on the bacterial host lawn. The lytic patterns were observed after 24 h of incubation at 37°C [19].

## Results

### Prevalence of *Salmonella* from different sources

Fifty-one *Salmonella* isolates were recovered from positive samples from all sources included in this study. Samples from slaughterhouse A presented the highest *Salmonella* prevalence (27.5%), followed by those from stall A (17.6%), commercial farm B (15.7%), and stall C (13.7%). However, samples from commercial farm A indicated a relatively low *Salmonella* prevalence (1.9%) (Table-1). The highest prevalence of *Salmonella* was associated with serovar Kentucky (31.4%), followed by Typhimurium (17.6%), Agona, Corvalis, and Schwarzengrund. Serovars including Albany, Give, Mbandaka, Singapore, and Weltevreden indicated a lower prevalence than the above serovars (Table-1).

**Table-1:** Prevalence of major *Salmonella* serovars in the broiler production chain.

Serovars	Sources								No. of isolates (% prevalence)^a^

	Commercial farm		Free- range farm	Slaughterhouse		Stall			
		
	A	B		A	B	A	B	C	
Agona	-	-	-	-	1	2	1	1	5/51 (9.8)
Albany	-	-	-	-	-	1	-	-	1/51 (1.9)
Corvalis	-	-	-	1	-	1	1	2	5/51 (9.8)
Give	-	-	-	1	-	-	-	1	2/51 (3.9)
Kentucky	-	-	3	7	1	2	1	2	16/51 (31.4)
Mbandaka	-	2	-	-	-	-	-	-	2/51 (3.9)
Schwarzengrund	-	-	-	2	2	-	-	1	5/51 (9.8)
Singapore	-	-	-	3	-	-	-	-	3/51 (5.9)
Typhimurium	-	4	-	-	-	3	2	-	9/51 (17.6)
Weltevreden	1	2	-	-	-	-	-	-	3/51 (5.9)
No. of isolates (% prevalence)^b^	1/51 (1.9)	8/51 (15.7)	3/51 (5.9)	14/51 (27.5)	4/51 (7.8)	9/51 (17.6)	5/51 (9.8)	7/51 (13.7)	

a The prevalence of each serovar found in all sources.

b The prevalence of *Salmonella* found in each source

### Prevalence of CIP-resistant *Salmonella*

CIP-resistant profiles were obtained from screening 51 *Salmonella* isolates (Table-2). Of these tested, 14/16 isolates (87.5%) of Kentucky and one isolate (50%) of Give showed to be CIP-resistant, whereas the other eight serovars were susceptible. Overall, the prevalence of CIP-resistance in *Salmonella* recovered from all sources included in this study was 29.4%. Overall, free-range farms, slaughterhouse A, and stall C were the predominant sources associated with the distribution of CIP-resistant *Salmonella*. Interestingly, no resistance was found in isolates collected from both commercial farms.

**Table-2:** Prevalence of ciprofloxacin-resistant *Salmonella* distributed in the broiler production chain.

Serovars	Sources								No. of resistant isolates (% prevalence)^a^

	Commercial farm		Free- range farm	Slaughter house		Stall			
		
	A	B		A	B	A	B	C	
Agona	-	-	-	-	-	-	-	-	0/5
Albany	-	-	-	-	-	-	-	-	0/1
Corvalis	-	-	-	-	-	-	-	-	0/5
Give	-	-	-	-	-	-	-	1	1/2 (50.0)
Kentucky	-	-	3	6	1	1	1	2	14/16 (87.5)
Mbandaka	-	-	-	-	-	-	-	-	0/2
Schwarzengrund	-	-	-	-	-	-	-	-	0/5
Singapore	-	-	-	-	-	-	-	-	0/3
Typhimurium	-	-	-	-	-	-	-	-	0/9
Weltevreden	-	-	-	-	-	-	-	-	0/3
No. of resistant isolates (% prevalence)^b^	0/51	0/51	3/51 (5.9)	6/51 (11.8)	1/51 (2.0)	1/51 (2.0)	1/51 (2.0)	3/51 (5.9)	15/51 (29.4)

a The prevalence of ciprofloxacin resistance *Salmonella* found in all sources.

b The prevalence of ciprofloxacin resistance *Salmonella* found in each source

### Isolation and lysis profiles of *Salmonella* phages

Eleven *Salmonella* phages were recovered from different wastewater and water samples. One phage (WPX4) was isolated from wastewater collected from the broiler farm. Two phages (WPX6 and WPX13) and one phage (WPX12) were recovered from the wastewater sediment and aerated treatment pond of the Prince of Songkhla University student dormitory wastewater treatment station, respectively. Three phages (WPX5, WPX10, and WPX11) and two phages (WPX8 and WPX9) from the sediment and aerated treatment pond of the wastewater treatment station of Prince of Songkhla University hospital, respectively. In addition, two phages were isolated from water samples of a natural reservoir (Table-3).

**Table-3:** Characteristics of *Salmonella* phages isolated in this study.

Properties	*Salmonella* phages										

	WPX4	WPX5	WPX6	WPX7	WPX8	WPX9	WPX10	WPX11	WPX12	WPX13	WPX14
Host of isolation	A4-525	S1H28	A4-525	A4-525	H2-016	H2-016	H2-016	H2-016	H2-016	H2-016	H2-016
Source of isolation	W1	W4	W2	NR	W5	W5	W4	W4	W3	W2	NR
Plaque morphotype (mm)	0.5	1.0	8.0	0.1	3.0	0.5	2.5	0.5	1.0	0.2	1.0

**Figure-1:**
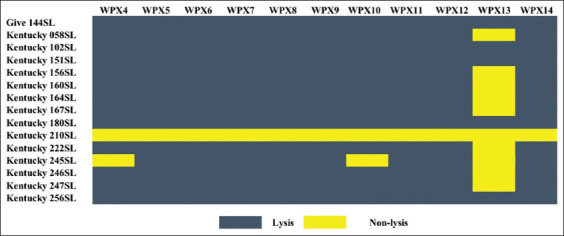
Lysis profiles of *Salmonella* phages on 15 ciprofloxacin-resistant *Salmonella* recovered from broiler production chain (blue area indicates lysis and yellow area indicates no lysis).

All selected phages showed different plaque morphotypes, ranging from a tiny 0.1 mm to a large plaque size of 8.0 mm (Table-3). Our phages exhibited a percentage lysis ability ranging from 33.3% to 93.3% in 15 CIP-resistant isolates (Figure-1). Most phages (8 of 11 phages) showed high lysis ability (93.3%), followed by two phages, including WPX4 and WPX10, which indicated a lysis ability of 87.6%. Only one phage (WPX13) exhibited the lowest lysis (33.3%). In this study, only one *Salmonella* isolates, namely 210SL, previously isolated from stall C, showed resistance to all phages tested.

## Discussion

Different samples from poultry farms, including bedding materials, feed, water, cloacal swab, boot cover swab, dust, and carcasses, have been reported as common sources of *Salmonella* [20]. In addition, poultry processing environments such as food contact surface, nonfood-contact surface, or equipment have been indicated to have *Salmonella* contamination [18]. Overall, *Salmonella* found in both the preharvest level (farm) and postharvest level (mainly slaughterhouse and processing plant) could be linked to the contamination of poultry meat products [21, 22]. Ziyate *et al*. [23] reported that *Salmonella* serovars, including Agona, Amsterdam, Enteritidis, Infantis, Kentucky, Thompson, and Typhimurium, were the significant serovars distributed in a poultry farm in Morocco, whereas *S*. Kentucky was frequently isolated from broiler farms located in Bangladesh [24] and Nigeria [25]. Other serovars, including Haifa, Kentucky, Saintpual, and Typhimurium, were found on broiler farms in Ethiopia [26]. In Thailand, common serovars including Albany, Agona, Corvalis, Enteritidis, Give, Kentucky, Mbandaka, Schwarzengrund, and Typhimurium were predominant serovars found in broiler farms, free-range farms, slaughterhouses, and stalls from the wet market [18, 19, 27, 28]. Similar to a previous report, *S*. Kentucky was the primary serovar contaminated in all sources included in our study (farm, slaughterhouse, and wet market). On a wet market, *S*. Agona, *S*. Corvalis, and *S*. Typhimurium were the predominant serovars found, whereas *S*. Mbandaka, *S*. Typhimurium, and *S*. Weltevreden were the predominant serovars detected in commercial and free-range farms. For the slaughterhouses, *S*. Schwarzengrund and *S*. Singapore were the most commonly detected.

The result of CIP resistance in *Salmonella* in our study is consistent with that of a previous report, which showed a lower frequency in commercial broiler farms (19%; interquartile range [IQR] 0.6%–40.1%) than other fluoroquinolone drugs (nalidixic acid, 80.3%; IQR 43.6%–97.6%) and ampicillin (64.8%, IQR 17.7%–92.1%). However, CIP resistance was highly observed in typical *Salmonella* serovars related to food contamination and human illnesses, including Enteritidis, Kentucky, Infantis, Typhi, Typhimurium, and Virchow [29–34]. Similarly, Xiong *et al*. [35] reported that up to 60.3% of *Salmonella* Kentucky isolated from the broiler production chain and patient specimens in China between 2010 and 2016 were identified as CIP-resistant because of the mutations in the quinolone resistance-determining regions and the presence of *Salmonella* genomic island 1. The wide spread of CIP resistance in *S*. Kentucky might be linked to the high prevalence of this serovar most commonly distributed in the broiler production chain worldwide, wherein this serovar has conferred multidrug resistance under antibiotic stresses [35–37]. To the best of our knowledge, there are no reports of CIP resistance in *S*. Give.

Phages are the most abundant biological entities on earth [38]. They have been commonly isolated from different types of water samples, especially wastewater and sewage [39]. Most phages also show diverse bacteriolytic patterns against many pathogenic bacteria [39–44]. *Salmonella* phages have also been recovered from wastewater and sewage [15, 19, 45]. The diversity of phage lytic activity depends on the source of phage origin. Some *Salmonella* phages exhibited a narrow host range or serovar-specific [46, 47], whereas others exhibited a diverse pattern of lytic ability [15, 19]. However, phages with a broad lytic activity are preferred to be used as a biocontrol to kill *Salmonella*. For example, the SEG5 phage could infect 16 of 22 (73%) *Salmonella* serovars tested [48]. *Salmonella* phages STm101 and STm118 isolated from Thai poultry farms could infect eight different *Salmonella* serovars commonly present in the poultry production chain [49]. In our previous study, vB_SenP_P32 phage isolated from a wastewater treatment pond exhibited the strongest ability to lyse up to 29 serovars of *Salmonella* (87.8%) [15]. Lytic phages induce bacterial cell lysis on endolysin action by destroying and breaking the peptidoglycan layer on the bacterial cell wall. Phages are continually reproduced after infecting the neighboring cells [38, 50]. Hence, the phage application in the food production chain is an attractive intervention because of the phage’s specificity, easy administration, and no harmful effect on human and animal health [51]. In this study, our isolated phages showed strong lytic activity against CIP-resistant *Salmonella* recovered from the broiler production chain included in this study. This suggests that their lytic activities are advantageous in combating CIP-resistant *Salmonella* strains in the broiler production chain and preventing their spread through the food supply chain. However, only one CIP-resistant isolate (SL210) was resistant to all phages tested. This might be because of the modification of phage receptors on the bacterial surface, restriction–modification systems, and/or adaptive immunity through the CRISPR-Cas system against phages [52–55].

## Conclusion

*Salmonella* phages obtained here provide useful information for designing the phage-based therapy for combatting CIP-resistant *Salmonella* in the poultry production chain. These phages can also be further applied to minimize the use of antibiotics in animal farming to reduce the emergence of CIP-resistant bacteria. Overall, phages are a potential alternative for controlling the spread of CIP-resistant *Salmonella* in broiler and other food production chains.

## Authors’ Contributions

WP: Designed the study, conducted the experiments, data analysis, discussion, and wrote the manuscript. AS, AK, and KV: Designed the study, participated in experiments, contributed to the conception, and funding. All authors have read and approved the final manuscript.
